# Supplementary figures and images for: A conserved transcription factor controls gluconeogenesis via distinct targets in hypersaline-adapted archaea with diverse metabolic capabilities
Source: PLoS Genet. 2024 Jan 16;20(1):e1011115. doi: 10.1371/journal.pgen.1011115 (PMC10817205; doi:10.1371/journal.pgen.1011115)

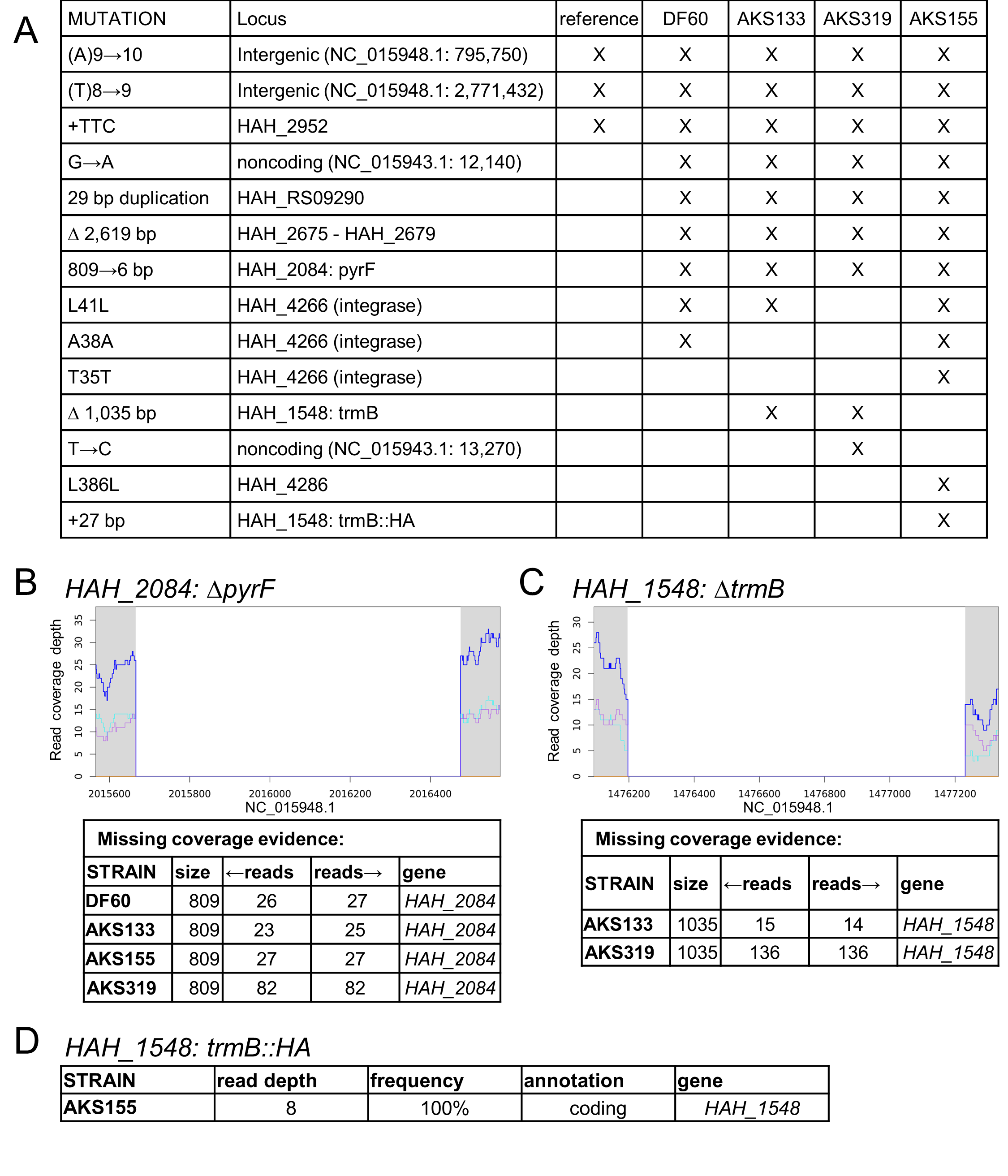

Supplement: S1 Fig — A: Summary of variants identified in each strain. “X” indicates that the mutation (rows) was detected in a given strain (columns). Strain designations are given in S1 Table. Representative coverage plots confirming chromosomal deletions for (B) pyrF locus for all strains and (C) trmBHar strains. X-axis provides the genome coordinates. Tables report local read depth, or sequencing coverage, for each strain. D: Confirmation of C-terminal trmB-hemagglutinin fusion used for immunoprecipitation experiments. (TIF) [file pgen.1011115.s001.tif]

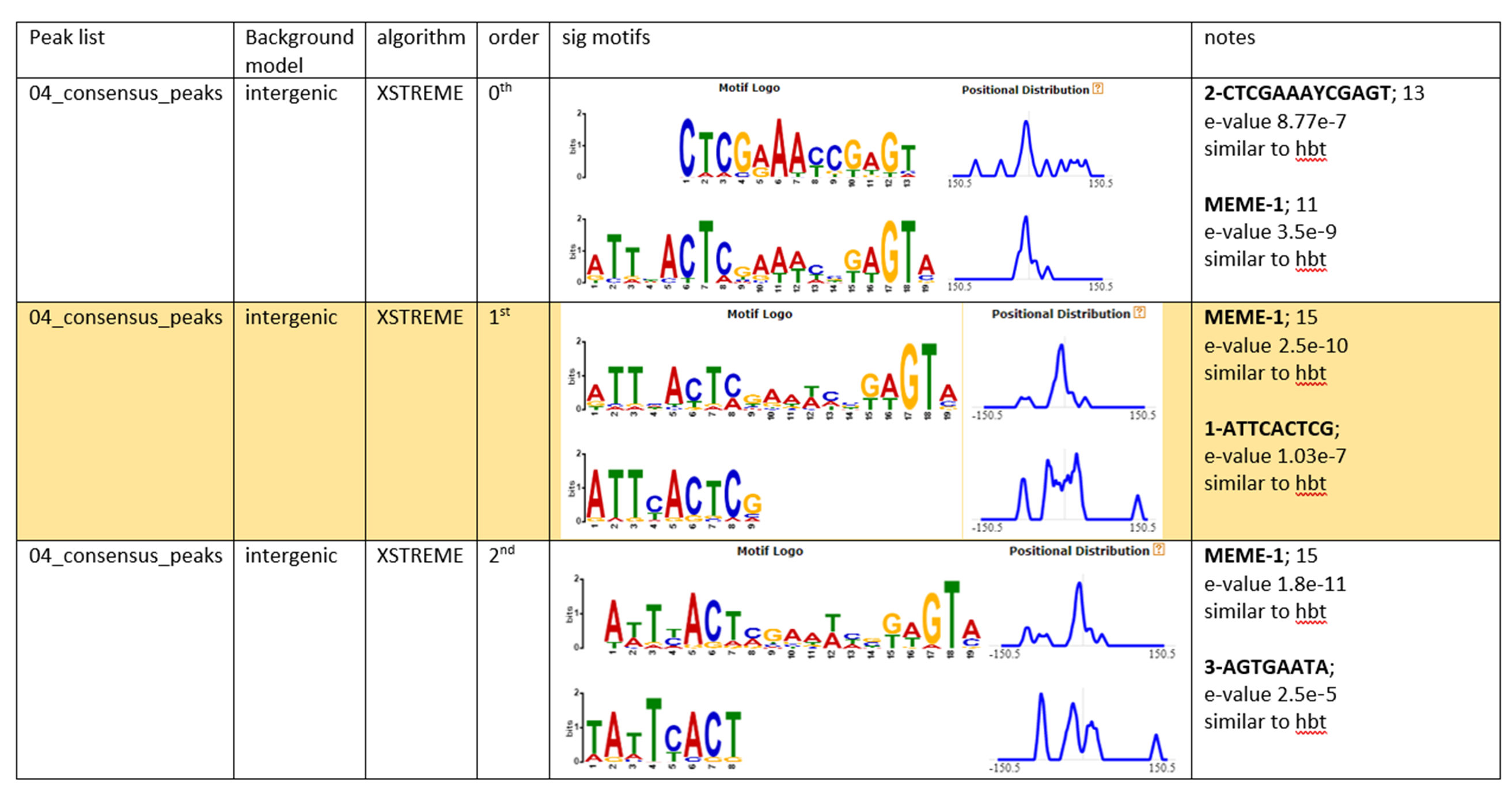

Supplement: S4 Fig — Sequences corresponding to peaks were extracted and submitted to XTREME as described in the methods. Yellow highlights indicate the motif reported in Fig 4. (TIF) [file pgen.1011115.s004.tif]

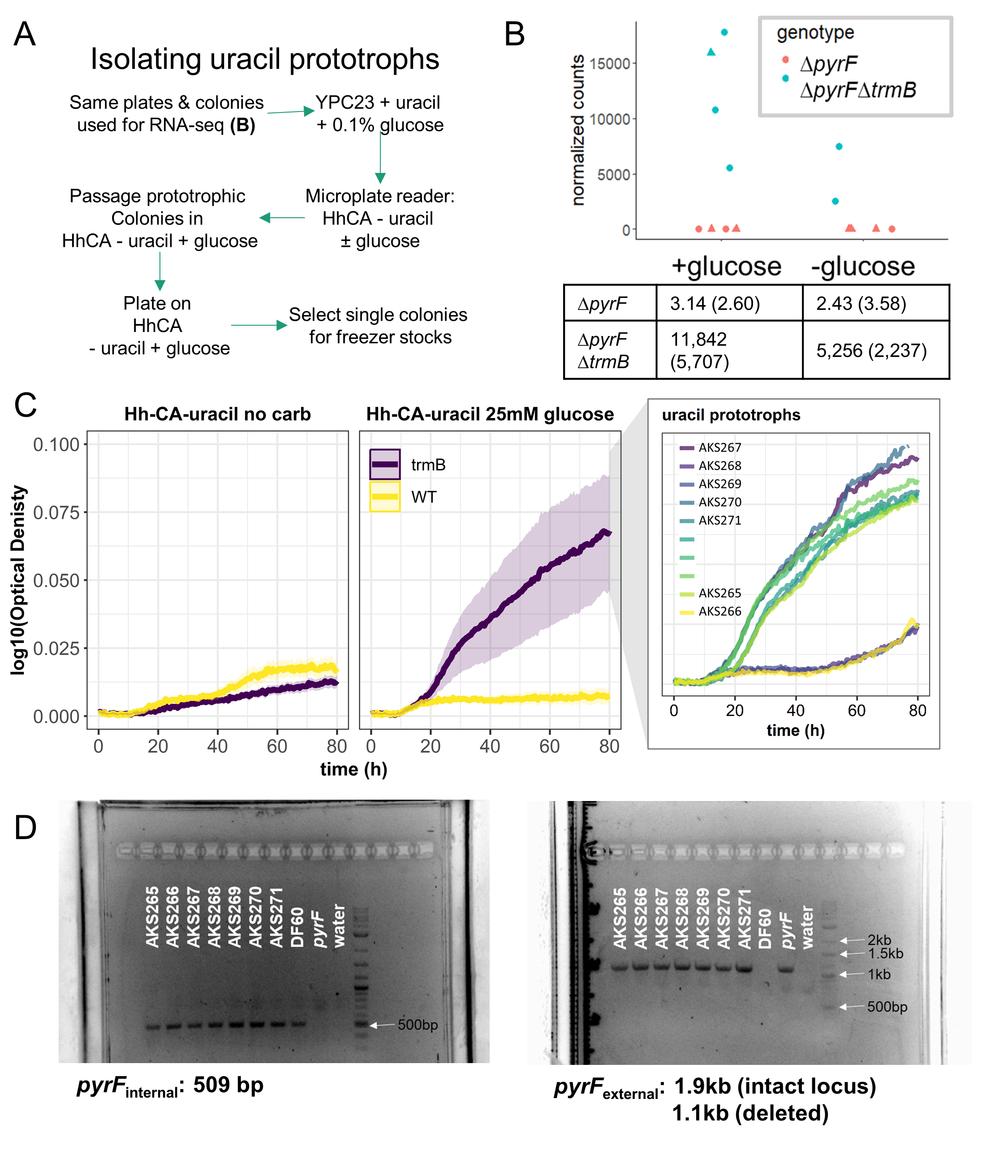

Supplement: S5 Fig — A: Diagram depicting the process of isolating uracil prototrophic strains from AKS133. B: Average number of transcripts mapping to pyrF in AKS133 RNA-seq samples. Standard deviation in parentheses. The point shape indicates the flow cell, or batch, on which the samples were sequenced. C: Log-transformed growth curves in the presence and absence of supplemental uracil and 5-FOA or no uracil. Shaded regions depict the 95% confidence intervals. Inset shows growth curves for individual cultures. Isolates AKS265–71 were obtained using the strategy summarized in A. D: Prototrophic isolates in C were streaked from freezer stock for genomic DNA extraction. Amplification by PCR indicates pyrF sequence is present in the genome (left), but that the endogenous deletion is intact (right). (TIF) [file pgen.1011115.s005.tif]

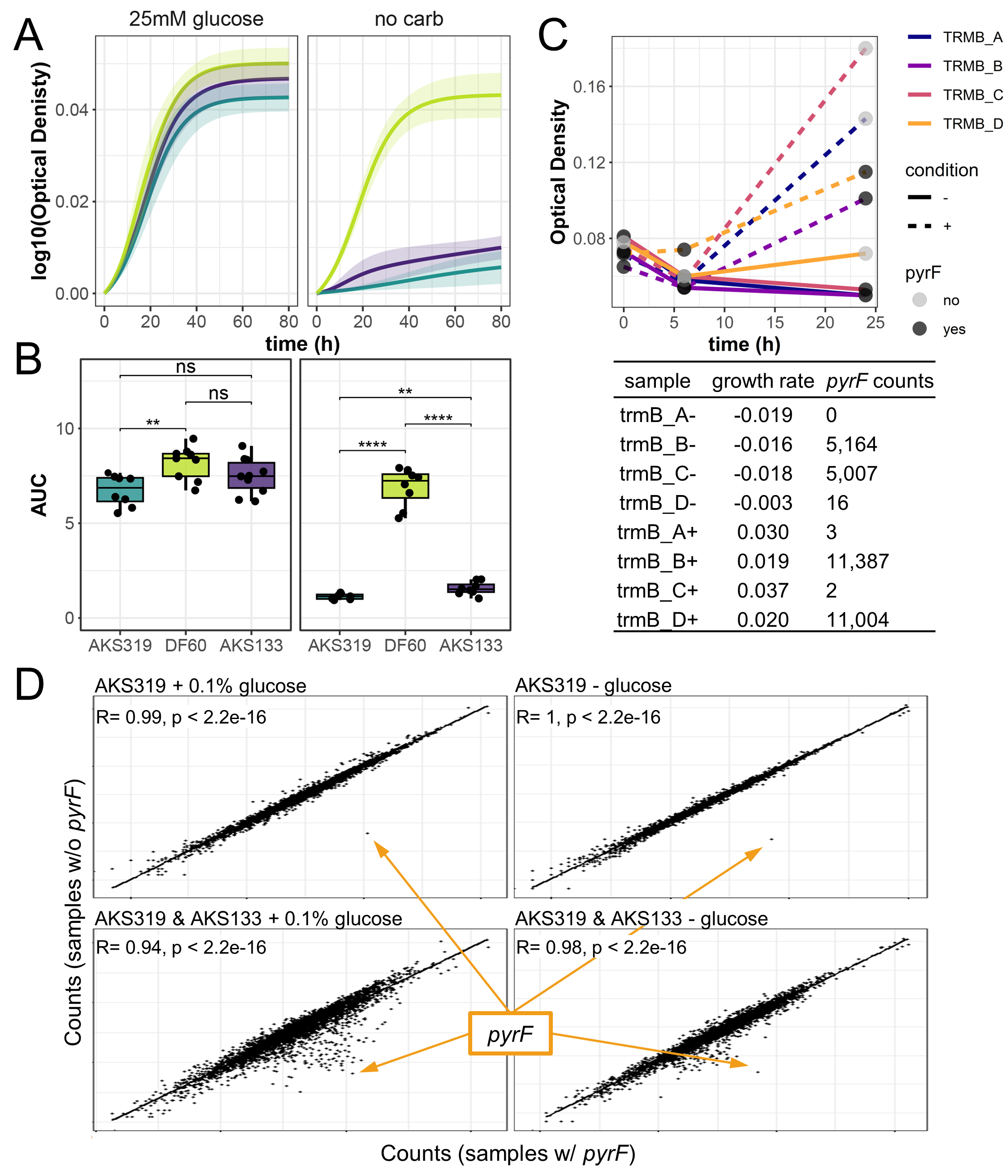

Supplement: S6 Fig — A: Fitted, log-transformed growth curves showing that AKS319 phenocopies AKS133, and (B) that there is no significant difference between AKS133 and AKS319 in 25 mM glucose as measured by the area under the curve (AUC). Strain colors are preserved in A and B. ** p-value < 0.01; **** < 0.0001. C: No significant differences in the growth rate of AKS319 cultures prior to RNA extraction between replicates exhibiting pyrF expression and not. Optical density measurements of the cultures harvested for RNA-seq are shown, with corresponding pyrF counts summarized in the table below. D: Average counts per transcript are highly correlated across AKS319 samples regardless of pyrF expression for both -glucose (N = 2) and +glucose conditions (N = 2). Average counts per transcript are highly correlated across AKS319 and AKS133 regardless of pyrF expression for both -glucose (N = 6) and +glucose conditions (N = 8). Average pyrF counts for each comparison are indicated in orange. Data are normalized relative to library size but have not been batch corrected. (TIF) [file pgen.1011115.s006.tif]

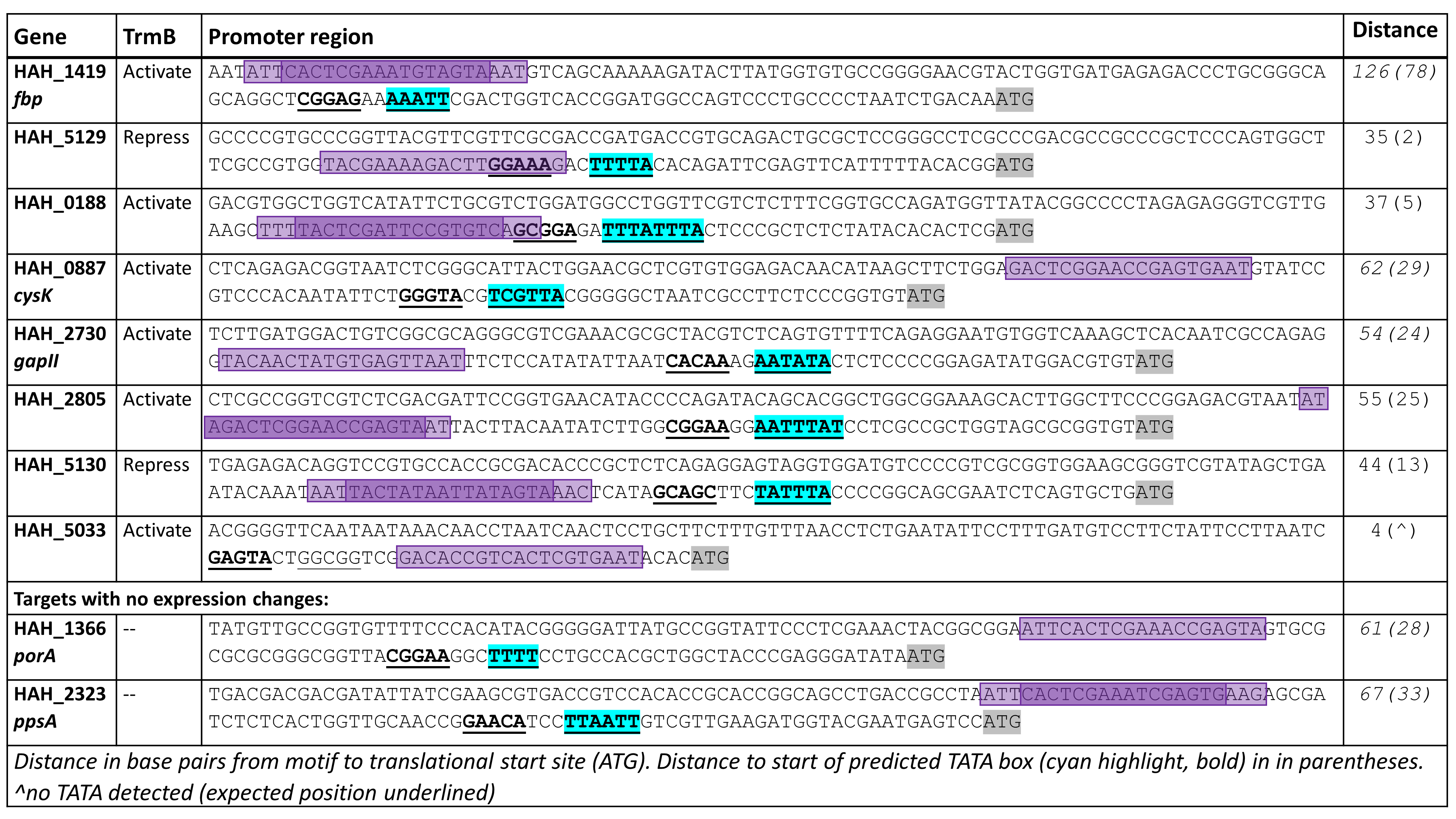

Supplement: S4 Table — Motif sequences are highlighted in purple. Motif occurrences on opposite strands were considered distinct. Darker purple color indicates motif instances on opposite strands overlap. Start codons are highlighted in grey. Putative initiation elements are bolded and underlined (TATA-box and BRE). Other haloarchaea have been reported to frequently lack identifiable TATA sequences [94]. If a promoter element could not be identified, the expected location (i.e., -26/-27 for TATA and -33/-34 for BRE) was underlined. (TIF) [file pgen.1011115.s013.tif]
