# Supplementary material for: A conserved transcription factor controls gluconeogenesis via distinct targets in hypersaline-adapted archaea with diverse metabolic capabilities
Source: PLoS Genet. 2024 Jan 16;20(1):e1011115. doi: 10.1371/journal.pgen.1011115 (PMC10817205; doi:10.1371/journal.pgen.1011115)
Supplement: S1 Table — (DOCX) [file pgen.1011115.s010.docx]

**Supplementary Table S1.** Strains used in this study

| Name | Species | Genotype | Purpose | Reference |
| --- | --- | --- | --- | --- |
| DF60 | *Haloarcula hispanica* ATCC33960 | Δ*pyrF* | growth assays, whole genome sequencing, ChIP-seq, RNA-seq | (1) |
| AKS133 | *Haloarcula hispanica* | Δ*pyrF* Δ*trmB* | Growth assays, whole genome sequencing, RNA-seq | This study |
| AKS155 | *Haloarcula hispanica* | Δ*pyrF trmB-HA* | ChIP-seq, whole genome sequencing | This study |
| AKS248 | *Haloarcula hispanica* | AKS133 *+* pAKS192 | TrmB-HA exogenous expression for complementation growth assays. | This study |
| AKS319 | *Haloarcula hispanica* | Δ*pyrF* Δ*trmB* | whole genome sequencing, RNA-seq | This study |
| AKS336 | *Haloarcula hispanica* | AKS319 *+* pAKS95 | TrmB exogenous expression for complementation growth assays. | This study |
| AKS265 | *Haloarcula hispanica* | Δ*pyrF* Δ*trmB* | AKS133 uracil prototroph isolated from well 53 | This study |
| AKS266 | *Haloarcula hispanica* | Δ*pyrF* Δ*trmB* | AKS133 uracil prototroph isolated from well 68 | This study |
| AKS267 | *Haloarcula hispanica* | Δ*pyrF* Δ*trmB* | AKS133 uracil prototroph isolated from well 125 | This study |
| AKS268 | *Haloarcula hispanica* | Δ*pyrF* Δ*trmB* | AKS133 uracil prototroph isolated from well 138 | This study |
| AKS269 | *Haloarcula hispanica* | Δ*pyrF* Δ*trmB* | AKS133 uracil prototroph isolated from well 143 | This study |
| AKS270 | *Haloarcula hispanica* | Δ*pyrF* Δ*trmB* | AKS133 uracil prototroph isolated from well 148 | This study |
| AKS271 | *Haloarcula hispanica* | Δ*pyrF* Δ*trmB* | AKS133 uracil prototroph isolated from well 158 | This study |

**REFERENCES**

1. Liu, H., Han, J., Liu, X., Zhou, J. and Xiang, H. (2011) Development of pyrF-based gene knockout systems for genome-wide manipulation of the archaea Haloferax mediterranei and Haloarcula hispanica. *Journal of genetics and genomics = Yi chuan xue bao*, **38**, 261-269.
