# Supplementary material for: A conserved transcription factor controls gluconeogenesis via distinct targets in hypersaline-adapted archaea with diverse metabolic capabilities
Source: PLoS Genet. 2024 Jan 16;20(1):e1011115. doi: 10.1371/journal.pgen.1011115 (PMC10817205; doi:10.1371/journal.pgen.1011115)
Supplement: S2 Table — (DOCX) [file pgen.1011115.s011.docx]

**Supplementary Table 2.** Plasmids used in this study

| Name | Description | Species | Reference |
| --- | --- | --- | --- |
| pHar | integration vector; Amp^R^, *pyrF* | *Haloarcula hispanica* | Lui 2011. 10.1016/j.jgg.2011.05.003 (1) |
| pWL502 | derivative of pWL102; replaced Mev^R^ with *pyrF* marker | *Haloferax mediterranei* | Cai et al, 2012. doi:10.1128/aem.07114-11 (2) |
| pNBKO7 | Template for pAKS83 Mev^R^ cassette | *Halobacterium salinarum* | Wilbanks 2012. 10.1093/nar/gks063 (3) |
| pAKS139 | pHar with *trmB* flanking regions for deletion | *Haloarcula hispanica* | This study |
| pAKS76 | pHar with trmB locus at BamHI site | *Haloarcula hispanica* | This study |
| pAKS87 | pHar with trmB-HA fusion at BamHI site | *Haloarcula hispanica* | This study |
| pAKS83 | pWL502 based self-replicating plasmid with Mev^R^ gene for selection | *Haloarcula hispanica* | This study |
| pAKS95 | pAKS83-based construct carrying *trmB* under its native promoter at the EcoRI and HindIII sites. | *Haloarcula hispanica* | This study |
| pAKS192 | pAKS83-based construct carrying *trmB-HA* translational fusion under its native promoter at the EcoRI and HindIII sites. | *Haloarcula hispanica* | This study |

**REFERENCES**

1. Liu, H., Han, J., Liu, X., Zhou, J. and Xiang, H. (2011) Development of pyrF-based gene knockout systems for genome-wide manipulation of the archaea Haloferax mediterranei and Haloarcula hispanica. *Journal of genetics and genomics = Yi chuan xue bao*, **38**, 261-269.

2. Cai, S., Cai, L., Liu, H., Liu, X., Han, J., Zhou, J. and Xiang, H. (2012) Identification of the haloarchaeal phasin (PhaP) that functions in polyhydroxyalkanoate accumulation and granule formation in Haloferax mediterranei. *Applied and environmental microbiology*, **78**, 1946-1952.

3. Wilbanks, E.G., Larsen, D.J., Neches, R.Y., Yao, A.I., Wu, C.Y., Kjolby, R.A. and Facciotti, M.T. (2012) A workflow for genome-wide mapping of archaeal transcription factors with ChIP-seq. *Nucleic acids research*, **40**, e74.
