# Supplementary material for: A conserved transcription factor controls gluconeogenesis via distinct targets in hypersaline-adapted archaea with diverse metabolic capabilities
Source: PLoS Genet. 2024 Jan 16;20(1):e1011115. doi: 10.1371/journal.pgen.1011115 (PMC10817205; doi:10.1371/journal.pgen.1011115)
Supplement: S3 Table — (DOCX) [file pgen.1011115.s012.docx]

**Supplementary Table 3.** Primers used in this study.

| Primer name | Sequence (5’ 🡪 3’) | Purpose |
| --- | --- | --- |
| Hh_1548_up_F | TCCGCTAAGGTACCTCTAGAAGAAGCTTGGGGAACCGACGGCTACTATGC | trmB deletion, locus amplification for integrant tagging |
| Hh_1548_up_R | CTACAGCCGACAGTGTCAGTTCTCACTAGACATAGTGGAGCGTTTGCG | trmB deletion |
| Hh_1548_down_F | CGCAAACGCTCCACTATGTCTAGTGAGAACTGACACTGTCGGCTGTAG | trmB deletion |
| Hh_1548_down_R | AGGGCCCCTGCAGGTCGACTCTAGAGGATCCTCGCTCGCAGTGACCGC | trmB deletion, locus amplification for integrant tagging |
| Hh_trmBHA_F2 | gccggattatgcgTGACACTGTCGGCTGTAG | HA-tag insertion |
| Hh_trmBHA_R2 | acatcatacggataGTTCTCGGGCGGTTCGTC | HA-tag insertion |
| MevR_BamHI_F | AAAGGATCCGGGTGTGTACCTCCGCGTTCGTC | Mev^R^ template for pAKS83 |
| MevR_SmaI_R | AAACCCGGGTTACCGACCGAGTTCGGCGTGGG | Mev^R^ template for pAKS83 |
| Hh_PtrmB_EcoRI_F | AAAgaattcCCGCTATCTCATCGCTGAAC | trmB and trmB-HA complementation plasmids |
| Hh_trmB_HindIII_R | TTTaagcttTCAGTTCTCGGGCGGTTCGTC | trmB complementation plasmid |
| HA_HindIII_R | TTTaagcttTCACGCATAATCCGGCACATCA | trmB-HA complementation plasmids |
| Hh_trmB_F | ATGTCTAGTGACGACTTGGAAGC | Sanger sequencing, plasmid verification |
| pHAR_MCS_1 | GACAGTCCGCGAAACAGCTC | Sanger sequencing, plasmid verification |
| pHAR_F | ACGACTCCGGTGACGCGTTCTTCA | Sanger sequencing, plasmid verification |
| pHAR_R | CATGATTACGCCAGATATCAAATT | Sanger sequencing, plasmid verification |
| Hh_idr1_F | CTTCGGATCCAGGACGAGCAGTTTGACAGC | gDNA contamination PCR |
| Hh_idr1_R | CTGTGGATCCTTCGATGGTATCACCGACCT | gDNA contamination PCR |
| Hh_pyrFint_R3 | AACTGATTCAGTCGCTGTTTG | Amplify 509bp region of pyrF CDS |
| Hh_pyrFint_F4 | ATTCTCGACGACGAGGAAGG | Amplify 509bp region of pyrF CDS |
| Hh_pyrF_PCR_F | CGACTCGGCTCGGCAATA | Amplify over the endogenous pyrF locus |
| Hh_pyrF_PCR_R | CCAGCATTCCGAGTATCCA | Amplify over the endogenous pyrF locus |
