# Supplementary material for: A conserved transcription factor controls gluconeogenesis via distinct targets in hypersaline-adapted archaea with diverse metabolic capabilities
Source: PLoS Genet. 2024 Jan 16;20(1):e1011115. doi: 10.1371/journal.pgen.1011115 (PMC10817205; doi:10.1371/journal.pgen.1011115)
Supplement: Attachment — Submitted filename: 2023-12-05-HCATrmB-PLOSGen-response to reviewers-AKS.docx [file pgen.1011115.s014.docx]

**PLOS Genetics D-23-00973 response to review**

We thank the reviewers for their helpful suggestions about this manuscript. In response, we have clarified our ChIP-seq methods, added a metabolic pathway diagram to clarify the introduction (new Figure 1), and made minor edits to the text and other figures / tables. From our perspective, these edits have considerably improved the manuscript but have not changed our original results or conclusions. Below, we respond point-by-point to specific reviewer comments, with reviewer and editor comments given in italicized text, and our responses in plain text.

**Reviewer's comments to the Authors:**

**Reviewer #1:** *Hackley et al characterise the function of the transcription regulator TrmB in the saccharolytic haloarchaeon* Haloarcula hispanica *using ChIP-seq to determine TrmB binding sites combined with RNA-seq and characterisation of growth phenotypes of a trmB deletion strain. The study is designed to compare the role of TrmB in* H. hispanica *to its homologue in* Halobacterium salinarum *that uses glycolysis pathways solely for gluconeogenesis and provide inside into the evolution of a transcription regulatory network. The authors show a more limited role of TrmB in* H. hispanica *where type II GAPDH and a fructose bisphosphatase (along sugar transporters) appear to be the key enzymes involved in gluconeogenesis via EMP that are regulated by TrmB transcriptionally. The experiments are carefully carried out and interpreted and besides one possible small issue with the data analysis I have only minor comments.*

**Major:**

- *Figure S3 is meant to visualise the ChIP-seq DNA fragment coverage. For a single binding sites of a transcription factor the ChIP-seq data would be expected to form a single peak over the binding site. Instead, Figure 3 shows consistently double peaks flanking the proposed binding site motif. This very much looks like read coverage (rather than DNA fragment coverage) where reads mapping on the + strand will pile up left of the binding site and reads mapping to the – strand on the right. The sequencing reads might not have been properly extended to the corresponding fragment size, or the estimated average DNA fragment size (150 bp) is incorrect and too short. The authors should revisit the code and ensure that this did not affect any other part of the data analysis.*

We sincerely appreciate the depth to which this reviewer analyzed our figures and methods relating to the ChIP-seq experiment and motif prediction. To clarify the point raised here, we did not use extended read files to generate Fig S3 (though extended bam files were used to generate Fig 3A in the main text). Our intention with this supplemental figure was to demonstrate that the identified peaks are also apparent in the raw data. We therefore decided to keep the original version of figure S3 rather showing than extended reads based on this motivation. However, we have clarified the figure S3 caption provide an example peak generated from extended read files (see figure below):

More generally, we’d like to clarify that two distinct methods of peak identification applied in this study. The first mode relied on prebuilt R packages mosaics, ChIPQC, and DiffBind, and we supplied unextended bam files as input. Separately, we also identified peaks using the per base enrichment across the genome as described in Grunberger *et al** with reads extended to 150bp in length. The reported 15 binding sites were identified in both analyses, thus we are confident in our downstream interpretation. Peak summits may shift slightly, but all identified binding sites are truly represented in the raw data. We have added a statement to the Methods to clarify (line 214).

* Grunberger F, Reichelt R, Waege I, Ned V, Bronner K, Kaljanac M, et al. CopR, a Global Regulator of Transcription to Maintain Copper Homeostasis in Pyrococcus furiosus. Front Microbiol. 2020;11:613532.

**Minor:**

- *Line 12: “Instead…” maybe turn into “Instead of allosteric regulation, ..”, as it does not follow on directly from the sentence before (presence of novel allosteric regulation points in archaea), but rather the sentence before that (absence of allosteric regulation of FBPA and PFK).*

This suggestion has been incorporated in the updated version (line 12).

- *Line 23/Figure 1: While the statement that TrmB TFs are more abundant in haloarchaea is most likely true, the number of identified TrmB homologues should be stated alongside the number of genomes for each taxonomic group included in the search to provide a fair comparison because of obvious biases in the composition of the data base.*

There were 357 proteomes in our HMMER analysis. 157 were Archaea, 97 of which were Halobacteria. There are on average 3 TrmB homologs per Halobacteria proteome (standard deviation = 2.09), while non-haloarchaeal Archaea have 1.8 homologs on average (standard deviation = 1.13). Halobacteria have significantly more TrmB homologs/proteome than other Archaea (p-value = 6.55x10^-5^, one-sided Wilcoxin Test) and other Archaea and Bacteria (p-value < 2.2x10^-16^, one-sided Wilcoxin Test). We have added the list of homologs to File S1 and added proteome numbers and significance values to the relevant results portion of the main text (lines 275-278) to better support this motivating statement in the introduction.

- *Line 38, line 53, line 479 and elsewhere: pykA, ppsA, gapI, porAB: it might be better to provide the gene names directly next to the full name of the enzymes for clarity.*

We have incorporated this suggestion throughout.

- *Line 67: PHBV should be spelled out.*

We have updated the main text accordingly (line 81 of revised manuscript).

- *Line 85: “archaeal-type GAPDH”: the manuscript mentions several times different types of GAPDH, it might be worth having a sentence in the introduction that first clarifies the existence of different GAPDH types in archaea.*

We have expanded the description of gluconeogenesis in haloarchaea (lines 63-76), including a clearer introduction to *gapII* (lines 71-72). We have also added a figure summarizing central carbon metabolism (also including *gapII*) as suggested by Reviewer 2 (now figure 1).

- *Line 189: extending the peaks to 300 bp intervals seems somewhat excessive as the real binding site is certainly found in a much narrower range (as evident in the motif position in the intervals), a single bp overlap to merge peaks appears to be too generous under these conditions as it would possibly merge peaks with their summits being possibly ~300 bp apart from each other.*

We agree that the TF binding site is likely much smaller than the 300 bp range shown in Fig 3B. However, we are not “extending” peaks to 300bp. Rather, the interval is reflective of the resolution of our libraries (the average fragment length of completed libraries was 405 bp, with a standard deviation of 75 bp). Using a smaller interval risks overfitting our data. However, to address your query about whether a 300 bp interval merges distinct peaks, we repeated the analysis with a 200 bp interval (reflective of the maximum estimated fragment size of IP libraries, see Table A below in this document). Shortening the interval did not increase the number of binding sites identified in differential enrichment analysis, nor did it shift the detected summits. We therefore did not modify the analysis in the revised manuscript.

|  | **300bp interval** | | | | **200bp interval** | | | |
| --- | --- | --- | --- | --- | --- | --- | --- | --- |
| chr (NC_) | start | end | LFC | Summit | start | end | LFC | Summit |
| 015948.1 | 1358448 | 1358749 | -6.94 | 1358598 | 1358498 | 1358699 | -7.06 | 1358598 |
| 015948.1 | 168780 | 169081 | -6.66 | 168930 | 168830 | 169031 | -6.92 | 168930 |
| 015944.1 | 133665 | 133966 | -5.96 | 133815 | 133715 | 133916 | -6.08 | 133815 |
| 015948.1 | 2701035 | 2701336 | -4.65 | 2701185 | 2701085 | 2701286 | -4.83 | 2701185 |
| 015948.1 | 2635395 | 2635696 | -5.22 | 2635545 | 2635445 | 2635646 | -5.55 | 2635545 |
| 015948.1 | 1211382 | 1211683 | -4.69 | 1211532 | 1211432 | 1211633 | -4.74 | 1211532 |
| 015948.1 | 1315388 | 1315689 | -4.46 | 1315538 | 1315438 | 1315639 | -4.57 | 1315538 |

*Table A: Regions that are differentially enriched in no glucose versus glucose IP samples. 7 sites reported in Table 2 of the main text is shown on the left, and the regions identified when the interval was reduced to 200 bp are shown in light grey. Position of peak summits are unchanged.*

If multiple peaks were being condensed, we would expect the summit to shift as the interval was trimmed, except in the rare case where the consensus peak was centered between 2 equally enriched binding sites. As we did not detect any shift in the summits, we interpret that the identified sites each represent a single binding site. We agree that it would be beneficial to modify the overlap required to merge peaks between samples (currently 1 bp), but this is not yet possible in DiffBind.

- *Line 197: “extended to their fragment size”: This should be the estimated average fragment size because the true fragment size remains unknown for single-read NGS data. What does the value of 150 bp reflect? Is this an average across different ChIP-seq libraries.*

The reviewer is correct, and we appreciate this clarification. We have updated the wording to clarify that this is the estimated fragment size generated by the ChIPQC package (line 213 of revised manuscript). We show the estimated fragment size for each IP library in Table B below. These values, and the code used to estimate fragment size are accessible on the updated GitHub repository (https://github.com/hackkr/Har_hispanica_TrmB). The average of all IP samples is 150 bp (149.75, specifically), while the average fragment length of input libraries is slightly longer. The estimated fragment size of individual samples was provided when calling consensus peaks in DiffBind, but we used the average when extending reads for figure generation (Fig 3A of main manuscript).

| WT_IP_0glu_A | 104 |
| --- | --- |
| WT_IP_0glu_B | 213 |
| trmB_IP_0glu_A | 104 |
| trmB_IP_0.1glu_A | 167 |
| trmB_IP_0glu_B | 200 |
| trmB_IP_0.1glu_B | 103 |
| trmB_IP_0glu_C | 103 |
| trmB_IP_0glu_D | 204 |

*Table B: Fragment sizes calculated by CHIPQC.*

- *Line 220: “required st(r)and specific read orientation” The difference between the HiSeq data for ChIP-seq and the NovaSeq data for RNA-seq should be that bowtie should be used in single-read or paired-end mode, respectively. Both mapping data would be strand-specific.*

Thank you for bringing this error to our attention. We have updated the sentence to read “alignment by Bowtie2 was done using pair-end mode” (line 237-8).

- *Line 224: “Logarithmic change” should be log2-fold change, naming the base.*

Corrected at line 242.

- *Table 2, page 15: I find the RPKM values not informative. Rather some enrichment value relative to the local surrounding region (as in MACS2 peak caller output) or normalisation against the input would be more meaningful.*

The reported RPKM values are normalized relative to the input (RPKM_IP_-RPKM_input_) as described in the caption. However, we agree that this metric is not the most intuitive. We have updated this column to now reflect the Log2 fold enrichment of the IP relative to input as returned by DiffBind (score = DBA_SCORE_RPKM_FOLD in dba.count function), to be more consistent with the other LFCs reported in the table. The updated analysis scripts are available on the GitHub repository. The rank of the peaks has changed, but the overall identity and annotation is preserved.

- *Page 16, Fig. 3 legend: Some details on the SEA p-value would be helpful. SEA measures enrichment of a motif in a data set, rather than comparing two motifs. Also, in the main text TomTom is cited as method used to compare the two motifs (page 19).*

This was an error in our explanation of XTREME. The p-value displayed is actually generated using TomTom (as part of the XTREME workflow), and indicates that the identified *Har. hispanica* motif is statistically similar to the *Hbt. salinarum* motif. We have updated the methods, citations, and legend accordingly (Fig 4 caption, page 17).

- *Line 348: As stated above, the intervals of 300 bp seem to be a bit excessive and unnecessarily underestimates the observed enrichment of binding sites in intergenic regions. Narrower, more realistic intervals or indeed looking simply at enrichment of the corresponding motifs themselves in intergenic regions would in my opinion justified.*

As stated above, 300 bp is roughly representative of the resolution of our libraries. Furthermore, the 300 bp intervals were only used for motif discovery by XTREME. The genome was then scanned with the 19 bp discovered motif and our downstream analysis differentiated between those that were in genic or intergenic regions. Motif scanning was carried out with FIMO (line 261-263), and there were 91 motifs identified in intergenic and promoter regions (File S1). We are not clear about how these methods differ from what the reviewer is suggesting in the second part of their comment.

To satisfy our curiosity and, hopefully, that of the reviewer, we found only very slight differences between the discovered motifs from 300 bp ChIP intervals versus 200 bp ChIP intervals:

300 bp interval

200 bp interval

- *Line 367: Given that the motif is in part palindromic, I am not sure it is correct to count motif occurrences on different strands at the same position twice.*

This is a good point. Though the motif is partially palindromic, and this pattern is relatively apparent by visual inspection, it is not typically detected as such with FIMO. There were only 7 motifs detected as palindromes/overlapping with FIMO (thus contributing 14 to the 67 motif instances reported in line 383). Even these palindromes are not perfect: they overlap by 17 bps with a 2 bp overhang and have distinct significance scores (visible in Table S4). We therefore prefer to leave the text as it is, as the numbers reported can be easily verified in File S1, and interested readers can discriminate between overlapping motifs on their own. We have sorted File S1, sheet 9 (tab “FIMO_annotated”) by motif location instead of significance so it is easier to detect when FIMO has identified distinct and overlapping motifs.

- *Line 400: I do not understand this interpretation for the pyrF integration elsewhere that TrmBHar “may be essential”. It is not essential because you deleted it successfully…*

While all chromosomal copies of *trmB* were successfully deleted, we were not successful at completely curing the population of the deletion vector (a step required to complete the construction of the deletion). Further, we assume the subpopulation of cells retaining *pyrF* have some fitness benefit that results in detection of *pyrF* expression and DNA detection following culturing for RNA-seq. Thus, we interpret that the *trmB* gene is conditionally essential in *ΔpyrF* background and with the methods and media used in our lab. Anecdotally, we have conducted RNA-seq with one other transcription factor deletion strain in *Har. hispanica* and did not detect *pyrF* expression, suggesting that this phenomenon might be specific to the ∆*trmB* strain. We have left this statement but further qualified as “conditionally essential” (line 417).

- *Line 434: The proper paper for the BRE to cite would be Bell, Kosa, Sigler, Jackson 1999 PNAS*

We have corrected this citation (line 452).

- *Line 434-436: It would be fair to say that there seem to be also some deviations from the pattern of TrmB motif position and activation/repression of transcription in the examples of Table S4 (e.g. in HAH_0188, binding site overlapping with the BRE, transcription activated)*

Yes, this is a fair interpretation of our data. We have included a phrase to better indicate the variability in this trend (line 456). This variation is consistent with other bifunctional TF motifs in archaea (Peeters *et al.*, 2013)*.

* Peeters E, Peixeiro N, Sezonov G. Cis-regulatory logic in archaeal transcription. Biochem Soc Trans. 2013;41(1):326–31. doi:10.1042/BST20120312

- *Line 445: For an uncharacterised sRNA, I would not state that it is monocistronic without any further evidence.*

We have removed the word “monocistronic” (line 464 of revised manuscript).

- *Line 446: Which peak is between two convergent genes? Is this the peak at rank 4 in table 2? Arrows pointing towards each other in the “genomic context” column would be helpful.*

We were referring to the intergenic peak (#3 in Table 2, Fig 4C), which is between two convergent genes in the genomic context beyond the large intergenic region shown in Table 2 and Fig 4C. However, we have removed this detail from the text as it is not clearly depicted in our figures / tables, nor is it essential to understand the small, unannotated transcript. Nevertheless, we have updated the genomic context figure (Fig 4C) to illustrate strand orientation of genes near binding sites more clearly for all peaks.

- *Line 459: “has fewer binding sites”: maybe better “’we detected fewer binding sites” given that two different methods (ChIP-chip vs ChIP-seq) and different analysis pipelines were used, for two different organisms… at least to some extend the difference in binding sites could be shaped by the methods. The authors for example for example state that 58 genes close to 67 potential TrmB binding motif occurrences are significantly enriched in carbohydrate transport and metabolism (Line 366). If this is true and not solely reflects the TrmB binding sites experimentally identified in ChIP-seq, then there could be additional TrmB binding sites that were beyond the sensitivity of ChIP-seq experiment.*

We have incorporated this change (line 477) and appreciate your point about the different methods used between species. To the best of our understanding, ChIP-seq offers increased resolution, specificity, and typically identifies more binding sites compared to ChIP-chip (Ho *et al.*, 2011)*, so we are less concerned with technical biases contributing to the fewer targets identified in *Har. hispanica*.

As you point out, it is possible that we did not detect all TrmB binding sites, particularly those that might be transiently bound early after glucose depletion. There are 5 genes in addition to *gapII*, *ppsA*, and *fbp* involved in carbohydrate transport/metabolism that have a motif in the promoters but no detected binding or differential expression, resulting in the functional enrichment reported. It is equally possible, given our current data, that these motifs are not functional/recognized by TrmB in *Har. hispanica*.

* Ho JW, Bishop E, Karchenko PV, gre N, White KP, Park PJ. ChIP-chip versus ChIP-seq: lessons for experimental design and data analysis. BMC Genomics. 2011;12:134

- *Line 493: It would be good to state the adjusted p-value for gapI and pgk to show the evidence for differential expression (even if not passing the significance threshold.)*

We have added the p-value at line 512.

- *Figure S5 legend panel B: What is the meaning of the triangles and dots?*

Samples were split (unequally) over 2 flow cells. The shape of the points indicated which flow cell the sample was sequenced on. Numbers reflect normalized, but not batch-corrected, counts. We have added this to the figure legend.

- *Supplementary Tables 1 and 3 are switched around*

This has been corrected.

- *Figure 2A: Multiple testing correction should be applied*

The growth difference tested is between the deletion strain and WT for each condition, not the ratios across conditions. It is identical to the statistical test shown in panel C (Fig 3 of revised manuscript), though the data are displayed differently. As such, it is not appropriate to correct for multiple testing. In the revised caption, we have clarified the statistical test applied.

- *Figure 3C: The y-axis should be -150 to +150 relative to peak position*

This has been corrected in the updated version of the figure (now 4C).

- *Figure 5C: The fbp gene is activated by TrmB, the purple colour of the arrow indicating transcription repression is wrong.*

Corrected (now Fig 6C in revised manuscript)!

**Typos/wording:**

- *Line 220 stand -> strand*

Corrected (this word was removed during editing in response to comments above, line 237-238 of revised manuscript).

- *Lines 381 and 386 pryF*

Corrected throughout.

- *Line 418: “TrmB-mediated induction” -> transcription activation?*

Updated to “consistent with transcriptional activation by TrmB” (line 436)

**Reviewer #2:** *The manuscript by Hackley et al describes a genomics and genetics study of the conserved TrmB transcription factor, and the role it plays in the saccharolytic haloarchaeal species Haloarcula hispanica. The study has been designed and conducted rigourously, and the data has been analysed and presented clearly. In my opinion, this manuscript requires only a few minor revisions before it is suitable for publication in PLoS Genetics.*

We thank the reviewer for their positive feedback.

***General point:*** *when reading the introduction, specifically the description of the glycolysis and gluconeogenesis pathways in line 44 onwards, I found myself searching for an illustration of these metabolic pathways. Just such an illustration then appears as Figure 5, but I feel that this is too late in the manuscript to be of help to a non-specialist. Consider moving Figure 5 (or at least the relevant pathways in Figure 5) to the introduction as Figure 1.*

We agree and have incorporated a diagram of central carbon metabolism in halophiles as Figure 1.

***Data query:*** *the authors find that the pyrF-marked deletion vector has repeatedly and independently integrated into the host genome by homologous recombination (lines 381-400). Would this not be evident in the whole genome sequencing data, given that the vector backbone should now be present? Do these sites of integration have anything in common? This information could be used to prevent integration by homologous recombination (e.g. by removing these sequences from the plasmid), which would be of interest to other Haloarcula genetics researchers.*

Thank you for this inquiry. We have not yet sequenced the genomes of the prototrophs, but there were no *pyrF* reads detected in the WGS of AKS319 or AKS133. We unfortunately do not, and the time of responding, have the complete pHar plasmid sequence to comprehensively investigate homology between the vector and genome. However, in response to this comment, we have submitted both the plasmid, AKS319, and one prototrophic revertant strain for long-read sequencing. We plan to share those results with the *Haloarcula* research community via the Halophiles email listserve once we have them.

***Literature:*** *in lines 350-352 it is stated that archaeal core promoter consensus sequences are still ill-defined, but in lines 432-434 the archaeal promoter architecture is described, with appropriate references. Consider moving this information and citation to lines 350-352.*

Line 367: We have updated this sentence to clarify that *Har. hispanica* promoters have not been experimentally characterized. Given this clarification, we left the details of the archaeal core promoter in place (lines 450-452 of the revised manuscript).

***Grammatical error:*** *throughout the manuscript, the term 'struck' is used to describe the streaking of cells from frozen stocks. The past participle of 'streak' is 'streaked', whereas 'struck' is the past participle of 'strike'. I appreciate that the term 'struck' is used widely in US laboratories, but it is not grammatically correct and risks confusing non-native English speakers*.

We have updated all instances of “struck” to “streaked”, and appreciate that this point was raised.

***Typographical error:*** *pyrF is occasionally misspelled as 'pryF', for example on line 381.*

We identified 5 instances of this typo and have corrected them. Thank you.
